# Variation of blubber thickness for three marine mammal species in the southern Baltic Sea

**DOI:** 10.3389/fphys.2022.880465

**Published:** 2022-11-23

**Authors:** Ursula Siebert, Miguel L. Grilo, Tina Kesselring, Kristina Lehnert, Katrin Ronnenberg, Iwona Pawliczka, Anders Galatius, Line A. Kyhn, Michael Dähne, Anita Gilles

**Affiliations:** ^1^ Institute for Terrestrial and Aquatic Wildlife Research (ITAW), University of Veterinary Medicine Hannover Foundation, Büsum, Germany; ^2^ MARE—Marine and Environmental Sciences Centre, ISPA—Instituto Universitário de Ciências Psicológicas, Sociais e da Vida, Lisbon, Portugal; ^3^ Department of Oceanography and Geography, Krzysztof Skóra Hel Marine Station, University of Gdansk, Hel, Poland; ^4^ Marine Mammal Research, Institute of Ecoscience, Aarhus University, Roskilde, Denmark; ^5^ German Oceanographic Museum, Stralsund, Germany

**Keywords:** blubber thickness, harbor seals, grey seals, harbor porpoises, natural variations, HELCOM area

## Abstract

Evaluating populational trends of health condition has become an important topic for marine mammal populations under the Marine Strategy Framework Directive (MSFD). In the Baltic Sea, under the recommendation of Helsinki Commission (HELCOM), efforts have been undertaken to use blubber thickness as an indicator of energy reserves in marine mammals. Current values lack geographical representation from the entire Baltic Sea area and a large dataset is only available for grey seals (*Halichoerus grypus*) from Sweden and Finland. Knowledge on variation of blubber thickness related to geography throughout the Baltic Sea is important for its usage as an indicator. Such evaluation can provide important information about the energy reserves, and hence, food availability. It is expected that methodological standardization under HELCOM should include relevant datasets with good geographical coverage that can also account for natural variability in the resident marine mammal populations. In this study, seasonal and temporal trends of blubber thickness were evaluated for three marine mammal species—harbor seal (*Phoca vitulina*), grey seal (*Halichoerus grypus*) and harbor porpoise (*Phocoena phocoena*)—resident in the southern Baltic Sea collected and investigated under stranding networks. Additionally, the effects of age, season and sex were analyzed. Seasonal variation of blubber thickness was evident for all species, with harbor seals presenting more pronounced effects in adults and grey seals and harbor porpoises presenting more pronounced effects in juveniles. For harbor seals and porpoises, fluctuations were present over the years included in the analysis. In the seal species, blubber thickness values were generally higher in males. In harbor seals and porpoises, blubber thickness values differed between the age classes: while adult harbor seals displayed thicker blubber layers than juveniles, the opposite was observed for harbor porpoises. Furthermore, while an important initial screening tool, blubber thickness assessment cannot be considered a valid methodology for overall health assessment in marine mammals and should be complemented with data on specific health parameters developed for each species.

## 1 Introduction

Evaluation of nutritional status (through distinct methodologies) has been frequently used in marine mammals as a proxy for the effects of environmental fluctuations on energy reserves at the individual level (Derous et al., 2020). Such evaluation is considered a good indicator tool to detect environmental signals, such as changes in the food supply (Heink and Kowarik, 2010). Variation in food supply may lead to adaptations in terms of life history traits (e.g., seasonal reproductive patterns) (IJsseldijk et al., 2021). When taking into account additional pressures originating from anthropogenic activities, such variations can lead to disruptions in marine mammals’ biological cycles, urging the need to closely monitor marine mammal populations (Kauhala et al., 2019).

Blubber thickness is regularly used to infer the nutritional status of marine mammals (Kauhala et al., 2019; Marón et al., 2021). However, it cannot be considered an accurate assessment of the nutritional condition of an individual as a standalone metric, since starvation processes include loss of fat (blubber thickness and lipid composition) as well as muscle mass (IJsseldijk et al., 2019). The blubber layer is one of the main energy storage sites in marine mammals, additionally providing insulation, supposedly functioning as a phase change material and contributing to buoyancy and hydrodynamic shape (Dunkin et al., 2005; Derous et al., 2020). Declines in blubber thickness have been associated with detrimental effects at the population level, such as reproductive failure and reduced survival rates (Spraker et al., 2020; IJsseldijk et al., 2021).

The Baltic Sea is an ecosystem exposed to massive impacts from anthropogenic activities resulting in cumulative pressures and potential threats to the sustainability of the wildlife occurring in the area (Reusch et al., 2018). Targeted surveillance schemes have been employed in the region to assess the status of several species groups, such as marine mammals, and inform management and mitigation strategies for these species (Backer et al., 2010).

Resident marine mammal species in the Baltic Sea (harbor seals, grey seals, harbor porpoises, and ringed seals; Kauhala et al., 2019) have different patterns of distribution and population structure. Under the Helsinki Commission (HELCOM), the convention for the protection of the marine ecosystem of the Baltic Sea, two harbor seal Management Units (MU) are recognized in the Baltic Proper; Kalmarsund and SW Baltic/Kattegat, although genetic studies have identified structuring within these units (Goodman, 1998; Olsen et al., 2014). Grey seals are recognized as one unit (Graves et al., 2009; Klimova et al., 2014; Fietz et al., 2016), distributed over the entire Baltic Sea. The population growth in later years has included a range expansion to the southern and western areas (Galatius et al., 2020). Regarding harbor porpoises, two distinct populations are recognized—the Baltic Proper population and the Belt Sea population in the western Baltic Sea, Belt Sea, the Sound and southern Kattegat (Tiedemann et al., 1996; Wiemann et al., 2010; Galatius et al., 2012; Lah et al., 2016). Considering this distributional and structural diversity, establishing standardized surveillance tools to be applied across the entire geographical area needs to be preceded by a solid knowledge of variation within and among the different management units/populations recognized in the Baltic Sea.

The main goal of the Marine Strategy Framework Directive (MSFD, 2008/56/EC) is to achieve Good Environmental Status (GES) in EU marine waters. GES is defined as a set of indicators representing different criteria (e.g., population size and demography), from which nutritional condition could be used as an indicator for demographic status, to assess the status of an environment/population (European Union 2008). Establishment of a blubber thickness metric for GES is complicated due to natural sources of variation including reproductive status, age, sex, and season. These sources of variation need to be accounted for. To this end, either a focused sampling of a random subset of the population at a specific time of the year (as under the current methodology according to HELCOM, 2018) with complementary full health status information or large datasets are necessary (Siebert et al., 2020).

In the Baltic Sea, monitoring programs implemented under HELCOM have established the evaluation of blubber thickness as a core indicator of nutritional status of marine mammals to assess the status of the marine environment (HELCOM, 2018). According to the surveillance scheme, and considering nutritional status alone, GES is achieved once recorded blubber thickness values in a sample of the population are above the determined threshold for individuals considered in a good condition. Some limitations exist, including data coverage with regard to species and geography. Until now, only grey and ringed seals have been included in this indicator concept of blubber thickness and GES thresholds have only been determined for grey seals, with no collated data available for harbor seals and harbor porpoises.

Currently GES thresholds for grey seals are only based on hunted and by-caught individuals, collected in Swedish and Finnish waters. Due to different water temperatures as a main trigger for variation in blubber thickness, with known variation from the Bothnian Bay to the southern parts of the Baltic Sea, possible geographical bias independent of variation related to the health status may be expected. However, it is relevant to first disclaim spatio-temporal variations of blubber thickness, as well as sex and age influence, in marine mammals in a certain geographical area prior to the implementation of thresholds to be used in wide populational assessments. In this study, we analyzed blubber thickness of the three marine mammal species occurring in the southern Baltic Sea—harbor seals, grey seals and harbor porpoises, based on records collected in dead animals.

## 2 Materials and methods

### 2.1 Animal collection and necropsy

Animals included in this study were collected by marine mammals’ stranding networks operating along the Baltic Sea coasts of Germany, Denmark and Poland, supplemented by regulated (shot) seals from Denmark. Harbor seals, grey seals and harbor porpoises found dead stranded along the coast or bycaught were transported to research facilities for a post-mortem evaluation. Animals were either assessed after admission or stored at −20°C for later analysis (depending on decomposition status). Necropsies were conducted on animals collected in Germany and Poland according to standardized protocols developed for these species, described by Siebert et al. (2001); Siebert et al. (2007).

During necropsy, sex was determined for each individual by evaluating the genital opening and the reproductive system. Age category was inferred either by determining the individual’s total length and the time of the year relative to the reproductive season. In single animals, age determination by evaluation of dental growth layer groups was conducted (Lockyer et al., 2010). The following age categories were considered: harbor and grey seals—*juveniles* (i.e.,; present and previous years’ offspring) and *adults* (i.e.,; animals older than 2 years, not identical with sexual maturity); harbor porpoises—*neonates* (i.e.,; animals younger than 6 months), *juveniles* (i.e.,; animals older than 6 months and younger than 4 years) and *adults* (i.e.,; animals older than 4 years) (Siebert et al., 2001; Siebert et al., 2007). Body measurements for all individuals included total weight, total length and body girth. On a subset of specimens, the individual health status was assessed based on pathological findings during necropsy, including state of muscles, and, when considered relevant, through additional testing (i.e.,; histopathology, microbiology, genetic and toxicology) (Siebert et al., 2001; Siebert et al., 2007; IJsseldijk et al., 2019; Siebert et al., 2020). Combined results from these investigations (no or mild lesions and infectious agents which can be considered “normal for a wild animal”) were considered to define health status. For animals collected in Denmark, the same measurements were recorded, but a thorough veterinarian necropsy was not conducted.

### 2.2 Blubber thickness measurements

**FIGURE 1:**
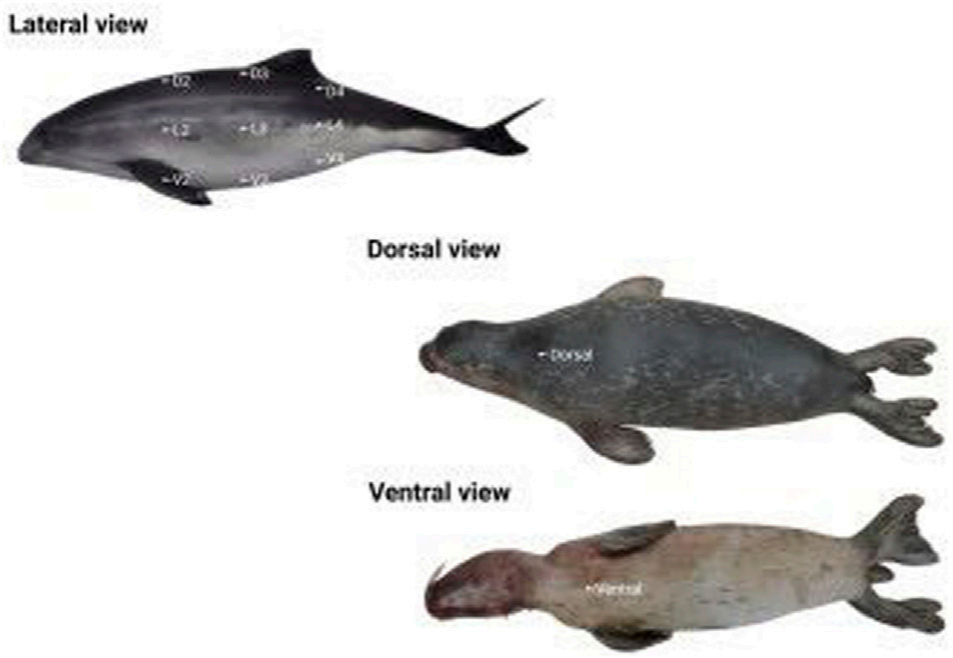
Locations of blubber thickness measurements: Lateral view for a harbor porpoise (*Phocoena*; D2, L2 and V2: cranially to the pectoral fin; D3, L3 and V3; D4, L4 and V4: caudally to the dorsal fin) and dorsal and ventral views dorsal blubber thickness measurement in a seal.

Blubber thickness was measured as described by Siebert et al. (2001); Siebert et al. (2007). Only carcasses displaying good to moderate decomposition status were selected for inclusion in the study (i.e.,; stages 1–3; IJsseldijk et al., 2019). This inclusion criterion was chosen in order to select cases where blubber thickness was not altered by autolytic processes, hence resembling thickness levels in live animals. Briefly, sectional cuts were made with a scalpel ranging from the skin to the muscle to expose the blubber in a vertical section without distortion. Measurements included the area starting at the interface between muscle fascia and the inner part of the blubber to the interface between the outer part of the blubber and the dermis. Blubber thickness was evaluated at different locations depending on the studied species. In harbor and grey seals this included two locations—1) on the ventral side, at the body’s midline of the front area of the thorax at the sternum; and 2) in the dorsal side, at the midline at the level of the center of the scapulae. In harbor porpoises, three sections were performed from the dorsal to the ventral side of the body—1) behind the pectoral fin (D2, L2 and V2), 2) in front (D3, L3 and V3) and 3) and behind the dorsal fin (D4, L4 and V4). At each section, a dorsal (D), a lateral (L) and a ventral (V) measure of the blubber thickness were recorded, comprising nine locations in total (Figure 1).

### 2.3 Statistical analysis

To evaluate the variance of blubber thickness for the three species, GAMs (generalized additive models) were applied, for each species, using blubber thickness (i.e., the mean of all measurement locations) as a dependent variable independently of the animals circumstances of death. The cyclic term “day of the year” and the “year” effect were included as temporal variables. It was hypothesized that differences between age groups and seasonal effects could occur, so the conditional variable “age group” was included. Regarding harbor porpoises, for the group of neonates, the term “day of the year” was not restricted to a cyclic term, as membership of this group does not apply for the entire year.

Moreover, sex and age group were included as factor variables. From a set of plausible candidate models backward model selection was applied to find the minimum adequate model based on AIC-values (see Table 1 for model selection and AIC-values). All analyses were run in the R environment 3.4 (R Core Team, 2017). GAMs were computed in the package mgcv (Wood, 2011).

**TABLE 1:** AIC-comparison of plausible models for the GAMs on blubber thickness for harbor seal, grey seal and harbor porpoises. Presented are the degrees of freedom (df), AIC-values and delta AIC in comparison to the minimum adequate model (in bold face). The variable ageandsex is a construction of age groups adult, juvenile (and neonates in the case of porpoises) differentiating between males and females only for the adults; → indicates symbol for conditional.

Model formula	df	AIC-value	Delta AIC
Harbour seals			
**sex + agegroup + s(year) + s(day of the year by age group)**	**10,848**	**1736,219**	**0**
sex + age group + s(year) + s(day of the year *→* age group) + s(day of the year *→* sex)	11,195	1736,568	0.349
ageandsex + s(year) + s(day of the year *→* ageandsex)	16,962	1737,347	1,128
age group + s(year) + s(day of the year → age group)	11,703	1743,068	6,849
sex + s(year) + s(day of the year *→* sex)	9,428	1815,827	79,608
Grey seals			
**sex + agegroup + s(day of the year by age group)**	**5,867**	**1330,911**	**0**
sex + age group + s(year) + s(day of the year *→* agegroup)	6,883	1332,817	1,906
ageandsex + s(year) + s(day of the year → ageandsex)	9,218	1332,595	1,684
age group + s(year) + s(day of the year *→* age group)	12,903	1338,127	7,216
Harbour porpoises			
**sex + agegroup + s(year) + s(day of the year by age group)**	**21,203**	**2263,791**	**0**
sex + age group + s(year) + s(day of the year)	13,214	2266,967	3,176
age group + s(year) + s(day of the year → age group)	11,226	2273,015	9,224
ageandsex + s(year) + s(day of the year *→* ageandsex)	17,153	2293,087	29,296
sex + age group + s(day of the year *→* age group)	6,221	2437,401	173,61

## 3 Results

In total, 1,037 harbor seals, 230 grey seals and 412 harbor porpoises were included in the analysis (Table 2). Harbor seal carcasses originated mostly from Denmark and Germany, in the period between 2002 and 2016. Grey seals were mostly collected from Germany, Poland and Denmark, between 1996 and 2016. Harbor porpoises originated mostly from Germany, Denmark and Poland, in the period between 1990–2016.

**TABLE 2:** Overview of samples included in the analysis (harbor seals: *n* = 1,307; Denmark—925, Germany—111, Unknown location—1; grey seals: *n* = 230; Germany—113, Poland—88, Denmark -28, Unknown location—1; harbor porpoises: *n* = 412; Germany—334, Denmark—55, Poland—13, Unknown location—10). A) Distribution of individuals over the collection period (harbor seals—2002–2016, grey seals—1996–2016, harbor porpoises—1990–2016). Juv, juvenile, Ad, adult, NA, Not attributed, Neon, neonate. B) Distribution of individuals over each quarter of the year, by sex and by age group. 1—January–March, 2—April-June, 3—July-September, 4—October-December. F—female, M—male, NA, Not attributed. Juv, juvenile, Ad, adult, NA, Not attributed, Neon.—neonate.

Species	Age	90	91	92	93	94	95	96	97	98	99	00	01	02	03	04	05	06	07	08	09	10	11	12	13	14	15	16	NA	Total
A																														
**Harbor seal**	Juv	—	—	—	—	—	—	—	—	—	—	—	—	87	1	3	10	11	3	6	1	13	8	4	14	22	12	3	0	198
	Ad	—	—	—	—	—	—	—	—	—	—	—	—	516	2	1	1	0	0	8	2	3	1	5	2	4	5	0	0	550
	NA	—	—	—	—	—	—	—	—	—	—	—	—	264	1	0	1	1	1	0	1	2	1	3	1	12	0	1	0	289
**Grey seal**	Juv	—	—	—	—	—	—	0	2	0	1	2	2	3	0	3	1	11	8	1	2	12	7	21	29	22	1	7	0	135
	Ad	—	—	—	—	—	—	1	0	2	0	4	3	8	2	2	0	0	3	3	2	3	7	11	9	21	3	10	0	94
	NA	—	—	—	—	—	—	0	0	0	0	0	0	0	0	0	0	0	1	0	0	0	0	0	0	0	0	0	0	1
**Harbor porpoise**	Neon	1	1	1	2	0	0	2	1	0	2	1	0	6	5	1	2	3	7	5	2	3	4	4	2	4	5	6	0	70
	Juv	5	17	2	5	7	4	9	4	7	1	15	9	6	14	5	10	13	13	18	12	2	11	10	7	4	14	22	0	246
	Ad	2	7	3	0	3	4	3	0	3	1	3	2	3	3	3	3	8	5	7	3	3	2	4	4	0	1	7	0	87
	NA	0	0	0	0	0	0	0	0	0	0	0	0	0	0	0	0	0	0	0	0	0	0	0	0	0	0	0	9	9

Quarter of the year								Sex
Species	Age	1	2	3	4	NA	Total	F	M	NA	Total
**B**											
**Harbor seal**	Juv	1	39	130	26	2	198	97	95	6	198
	Ad	5	63	360	116	6	550	210	288	52	550
	NA	1	69	154	60	5	289	123	131	35	289
**Grey seal**	Juv	5	108	16	4	2	135	63	66	6	135
	Ad	18	32	21	23	0	94	26	65	3	94
	NA	0	1	0	0	0	1	0	1	0	1
**Harbor porpoise**	Neon	1	3	57	7	2	70	26	44	0	70
	Juv	38	34	94	76	4	246	116	124	6	246
	Ad	8	19	33	26	1	87	53	32	2	87
	NA	0	0	0	0	9	9	0	0	9	9

### 3.1 Harbor seals

**FIGURE 2:**
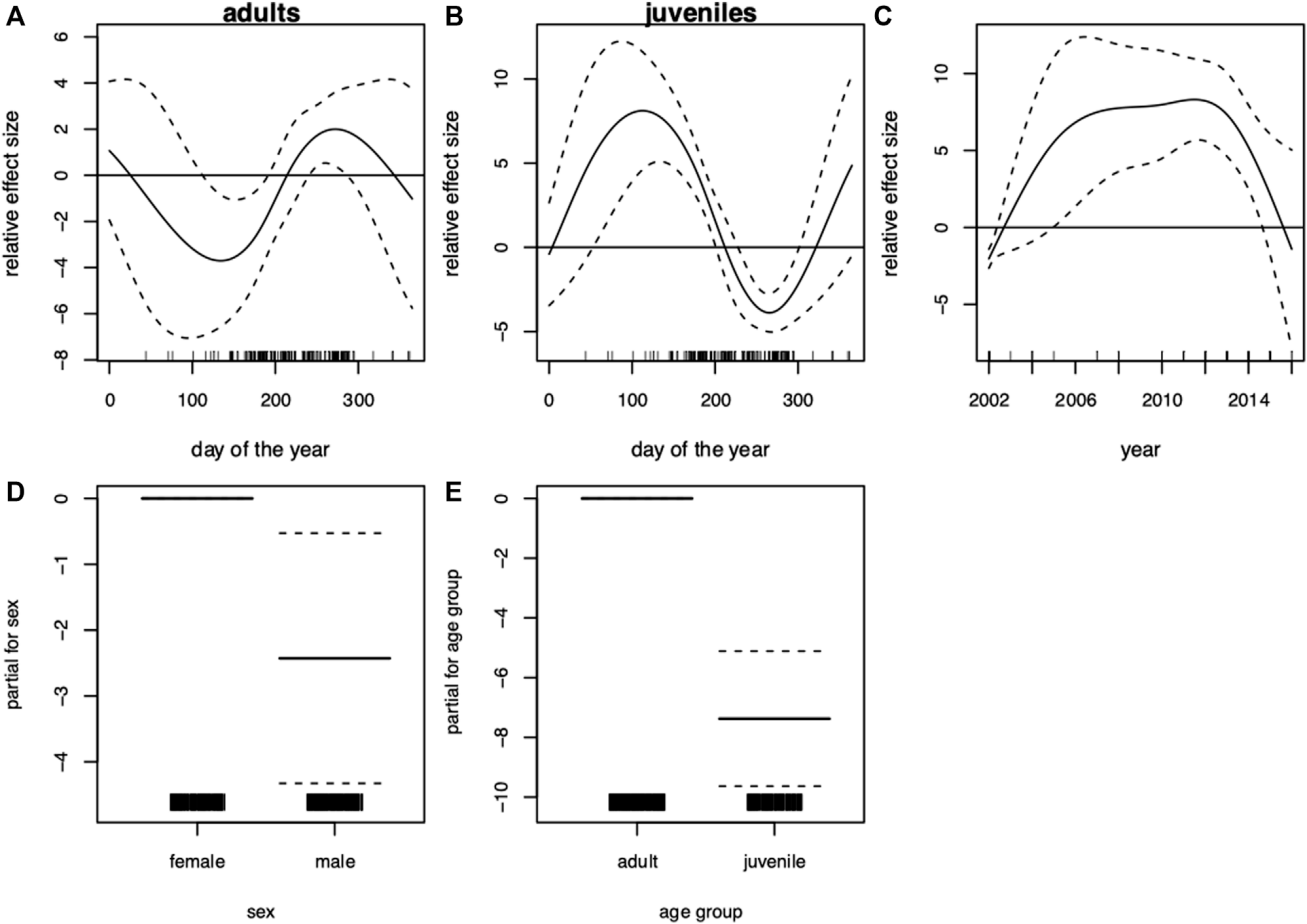
Harbor seal (*Phoca vitulina*), South-western Baltic Sea (incl. Kattegat). Generalized additive model (GAM) of blubber thickness showing the seasonal dependency (day of the year 1–365) conditional on age group **(A)** adults, **(B)** juveniles (present and previous years offspring) and the development over the **(C)** years. Moreover, the parametric terms of **(D)** sex and **(E)** age group. The *y*-axes represent the term’s spline function (solid line). Zero on the *y*-axes corresponds to no effect of the predictor variable. The dotted lines reflect two times the SE bands (i.e., 95% confidence interval). Hatch marks on the *x*-axes show sample values and ranges. Deviance explained = 40.9%, *n* = 252.

The results showed a wide variability of blubber thickness values depending strongly on season and age. The seasonal pattern of blubber thickness in adults was more pronounced (F-value of 16.9 compared to 2.8 in juveniles, Table 3). In adults, lowest blubber thickness values were recorded around the time of weaning and mating season (August—September). After this period, blubber thickness increased steeply (Figure 2A). The GAM of blubber thickness revealed seasonal effects in juveniles (Table 3). In this age group, lower blubber thickness values were recorded around the time of birth (April–July), while the highest values were recorded in the transition of late summer to early autumn (Figure 2B). During winter, sample size was too low resulting in wide confidence intervals; effects could therefore not be evaluated. Blubber thickness increased over the years included in the analysis, peaked around 2012 and then decreased (Figure 2C). In general, males presented thinner blubber layers than females (Figure 2D). Similarly, blubber layers in juveniles were thinner than in adults (Figure 2E).

**TABLE 3:** GAMs of blubber thickness as related to day of the year conditional on 1) harbor seals—age group juveniles (present and previous years offspring), adults and the development over the study period; 2) grey seals - age group juveniles (present and previous years offspring) and adults; 3) harbor porpoises - age group neonates, juveniles and adults. Moreover, the parametric terms of sex and age group, showing the significant effects of the fixed effects, are given (estimates, SE, *t*-values, and *p*-values); as well as the approximate significance of the smooth terms (the estimated degrees of freedom [edf], the estimated residual degrees of freedom [Res.df], and the F- and *p*-values). Deviance explained = 40.9% (harbor seals), 11.9% (grey seals), 47.2% (harbor porpoises); *n* = 252 (harbor seals), 171 (grey seals), 345 (harbor porpoises).

Parametric coefficients					
		Estimate	Std. Error	*t* value	Pr(>|t|)
**Harbor seal**	(Intercept)	14.5181	1.0046	14.452	<0.001
	male	−2.8662	0.9501	−2.555	0.011
	juvenile	−7.3750	1.1307	−6.523	<0.001
**Grey seal**	(Intercept)	23.918	1.97	12.144	<0.001
	juvenile	2.405	2.026	1.187	0.237
	male	3.777	1.876	2.014	0.046
**Harbor porpoise**	(Intercept)	18.1636	0.7676	23.663	<0.001
	male	−1.0214	0.6973	−1.465	0.144
	juvenile	6.8849	0.7543	9.127	<0.001
	neonate	2.0888	4.4232	0.472	0.637
**Approximate significance of smooth terms**					
		**edf**	**Res.df**	**F**	***p*-value**
**Harbor seal**	dayofyear: juvenile	1.595	3	2.798	<0.01
	dayofyear: adult	2.030	3	16.896	<0.001
	year	3.224	3.667	13.379	<0.001
**Grey seal**	dayofyear: juvenile	1.867	3	3.907	<0.001
	dayofyear: adult	0.000	3	0.000	0.374
**Harbor porpoise**	dayofyear: neonate	2.289	2.753	3.414	0.012
	dayofyear: juvenile	3.684	4.664	34.322	<0.001
	dayofyear: adult	2.794	3.587	9.229	<0.001
	s(year)	1.997	2.432	2.651	0.069

### 3.3 Grey seals

**FIGURE 3:**
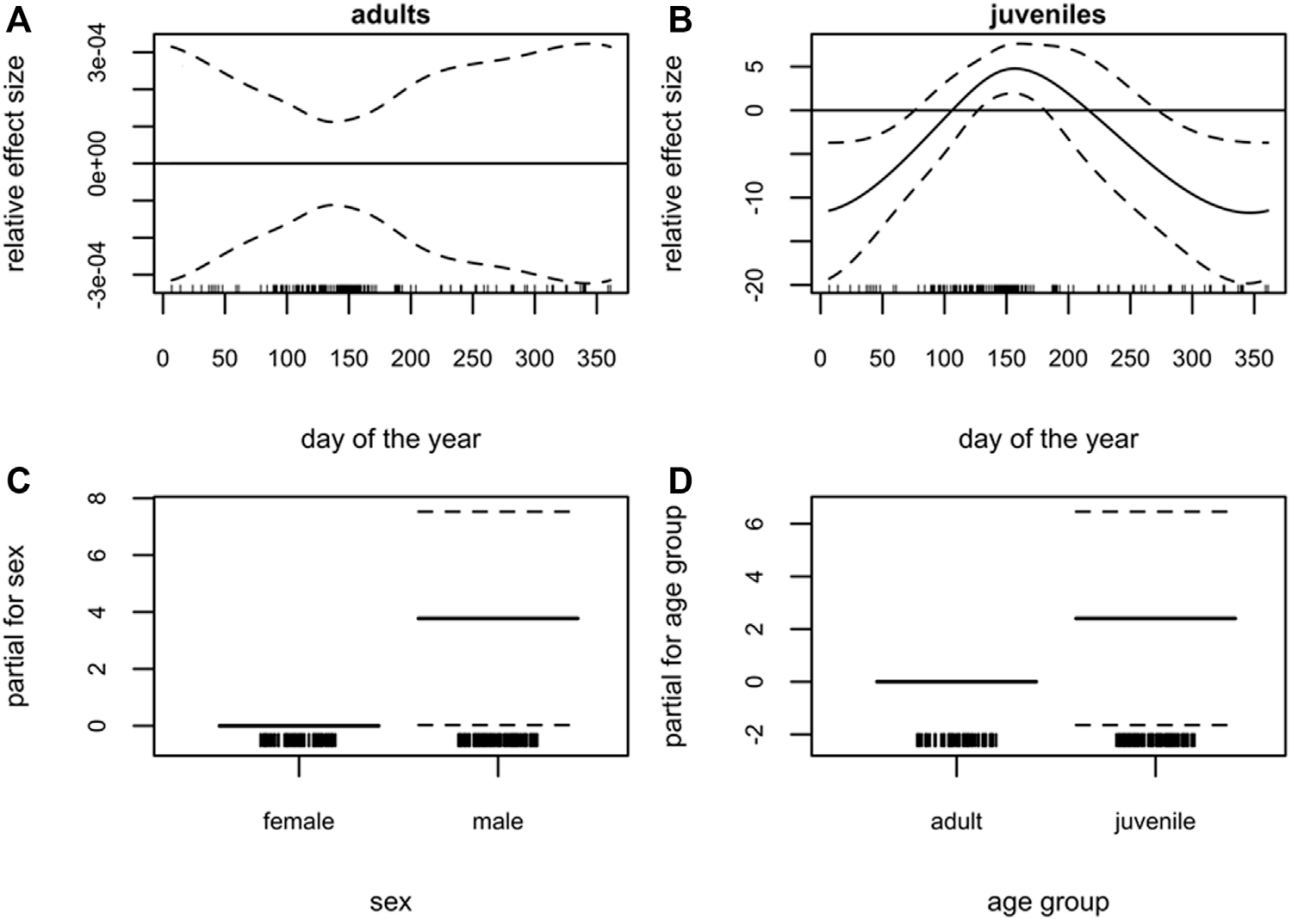
Grey seal (*Halichoerus grypus*), South-western Baltic Sea. Generalized additive model (GAM) of grey seal blubber thickness showing the seasonal dependency (day of the year 1–365) conditional on age group **(A)** adults, **(B)** juveniles (present and previous years’ offspring). Moreover, the parametric terms of **(C)** sex and **(D)** age group. The *y*-axes represent the term’s spline function (solid line). Zero on the *y*-axes corresponds to no effect of the predictor variable. The dotted lines reflect two times the SE bands (i.e., 95% confidence interval). Hatch marks on the *x*-axes show sample values and ranges. Deviance explained = 11.9%, *n* = 171.

In contrast to harbor seals, the GAM of blubber thickness using the grey seal samples explained much less of the deviance in the data (11.9%). The seasonal effect was not significant in adults (Figure 3A), whereas the seasonal pattern in juveniles was more pronounced (*p* < 0.01, Figure 3B). Blubber thickness in juveniles showed the highest values in June and lower values in December and January (Figure 3B). Furthermore, no differences could be detected between age groups (Figure 3D). Female grey seals, overall, had a thinner blubber layer than males (*p* = 0.046) (Figure 3C).

### 3.4 Harbor porpoises

**FIGURE 4:**
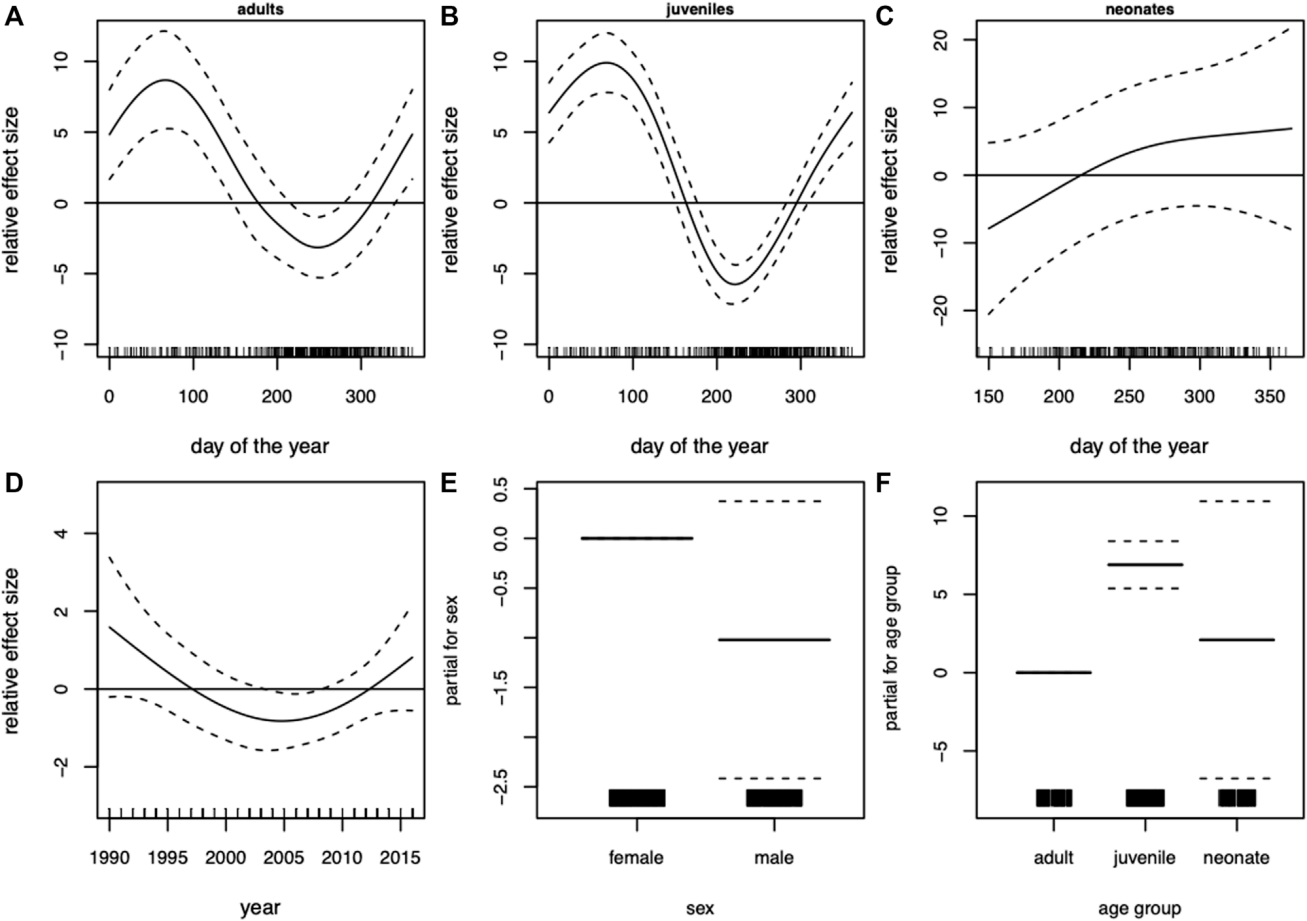
Harbor porpoise (*Phocoena*). Generalized additive model (GAM) of blubber thickness showing the seasonal dependency (day of the year 1–365) conditional on age group **(A)** adults, **(B)** juveniles, **(C)** neonates and **(D)** the development over the years. Moreover, the parametric terms of **(E)** sex and **(F)** age group. The *y*-axes represent the term’s spline function (solid line). Zero on the *y*-axes corresponds to no effect of the predictor variable. The dotted lines reflect two times the SE bands (i.e., 95% confidence interval). Hatch marks on the *x*-axes show sample values and ranges. Deviance explained = 47.2%, *n* = 345.

**FIGURE 5:**
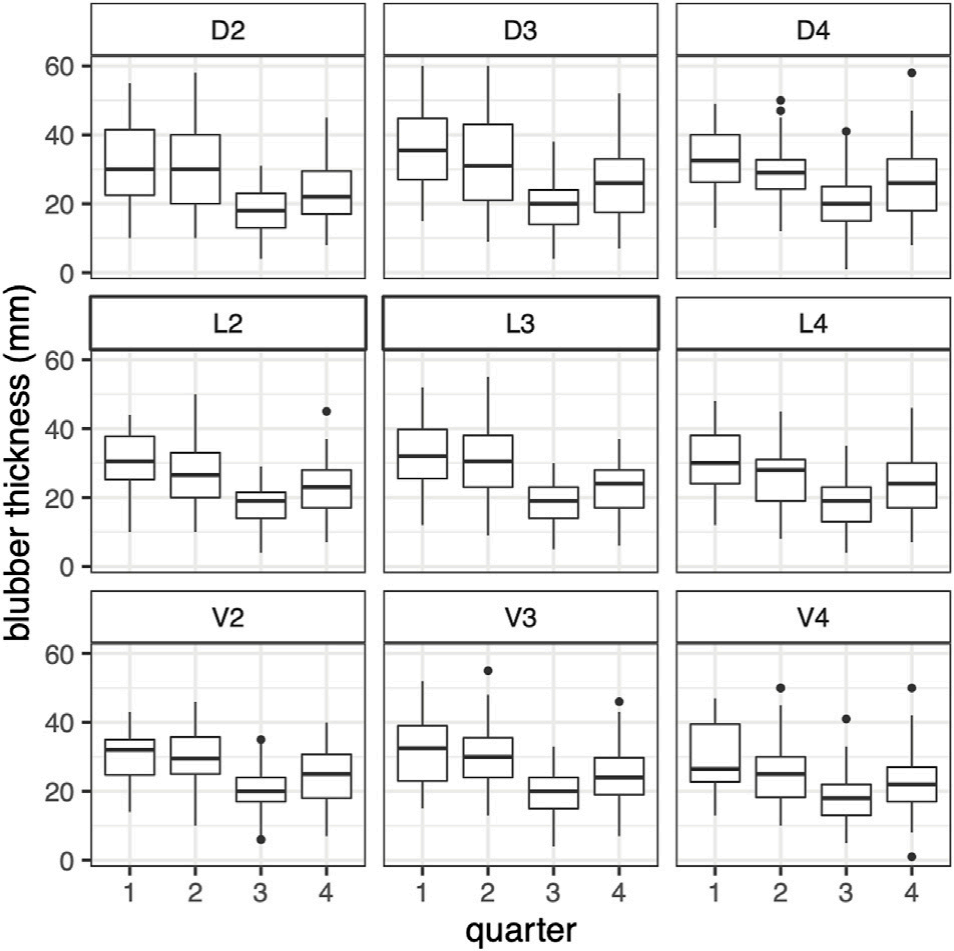
Harbor porpoises blubber thickness of 9 measurements points over all quarters (quarter 1 = January–March, 2 = April–June, 3 = July–September, 4 = October–December), shown for health status “good” (*n* = 151).

The GAM of blubber thickness in harbor porpoises showed a strong seasonal effect in adult individuals. Highest values were recorded in winter and spring and the lowest values in late summer and early autumn (Figure 4A). The seasonal pattern in juveniles was more pronounced (F-value of 33.7 compared to 10.9 in adults, Table 3). Nonetheless, the overall seasonal pattern was equivalent between both age groups (Figures 4A,B). A significant seasonal dependence of blubber thickness in neonates was observed (*p* = 0.008, Table 2), however, this effect was not very pronounced. In this age group, blubber thickness records increased over the relevant period of the year (a minimum value of 155 days for “day of the year”, the estimated time of birth, was set for neonates, restricting the x-scale to values above 155 days, Figure 4C). Blubber thickness was relatively constant over the years included in this analysis. However, a phase where lower blubber thickness values were recorded could be observed approximately from 2000 to 2010 (Figure 4D). Overall, male individuals had a thinner blubber layer than females, however, this effect was not significant (Figure 4E). Juveniles had a significantly thicker blubber layer than adults, whereas neonates showed no significant difference to juveniles and adults (Figure 4F). Furthermore, different measuring points on the body were tested for seasonal effects using quarters of the year. Results showed that the blubber layer varies similarly/in parallel at all locations over the year (Figure 5) and, thus, no preferable measuring point within these nine locations could be identified.

## 4 Discussion

Establishing indicator parameters to evaluate the general health of a population of marine mammals in order to make inferences about populational trends is challenging. The use of information collected in large datasets involving stranded individuals, such as those collected under joined efforts from stranding networks operating in a specific region, can help to evaluate the reliability of indicators and their further implementation in surveillance operations. Blubber thickness evaluation can provide important information about the energy reserves, and hence, food availability in the Baltic Sea. However, methodological standardization to be performed under HELCOM should include relevant datasets with good geographical coverage. It is relevant to highlight that the development of methodologies for environmental and biodiversity holistic assessments such as in HELCOM are not geographically limited to the Baltic Sea. Strengthening indicator criteria for a wider use in wild marine populations is also aimed by other Regional Sea Conventions (e.g., OSPAR; McQuatters-Gollop et al., 2022). Simultaneously, management guidelines are regulated by international conventions that expand beyond original geographical aims.

In this study, analyses of the variation of blubber thickness with regard to age, season and sex, as well as longer-term trends, were performed for the first time for harbor seals and harbor porpoises in the Baltic Sea, as well as for grey seals in the southern Baltic Sea. Our analyses detected pronounced fluctuations observed both in terms of season, year of sampling, sex and age category; confirming the importance of taking such parameters into consideration when using blubber thickness as an energy reserve indicator.

Our analyses provide a baseline for blubber thickness variation and dynamics for both juvenile and adult harbor seals in the southwestern Baltic Sea. Comprehensive investigations with ample samples are scarce in these species (Pitcher, 1986; Hall et al., 1999), but variability in reference values is evident among studies, even when samples are divided by age groups (compiled in Liwanag et al., 2012). Blubber thickness values are further influenced by seasonality, with a pronounced negative effect in the blubber thickness values caused by large energy expenditures in specific time spans related to reproduction and moulting and when more insulation is needed in winter. For harbor seals, periods of weight loss have been associated with the lactation period in females and the mating season in males (Härkönen & Heide-Jørgensen, 1990). Previous reports also found results similar to ours with respect to age categories and sex in harbor seals, with females and adults presenting thicker blubber layers (Pitcher, 1986).

As for harbor seals, information on blubber thickness values in grey seals is also limited (Nordøy and Blix, 1985; Folkow and Blix, 1987). For grey seals in the Baltic Sea, large data sets on blubber thickness thresholds is available for animals in the northern areas—Swedish and Finish waters (HELCOM, 2018). Blubber thickness fluctuates along with herring weight both spatially and temporally in Finnish and Swedish waters, thus providing evidence that it is related to food web processes in the Baltic Sea (Kauhala et al., 2017). From the proposed indicator threshold values based on these Swedish and Finnish data (40 mm for hunted seals and 35 mm for by-caught seals during fall, 25 mm for populations under effects of density dependence; HELCOM, 2018), it can be concluded that, at the population level, grey seals found in the Southern areas studied here do not currently achieve GES as defined in the HELCOM core indicator nutritional status. However, it is unclear on which baseline data these thresholds are based. Our results indicate that some variability in blubber thickness exists for grey seals in the southern Baltic Sea, not only regarding season, age category and sex, but also among animals in a good health status. This may translate into misjudgments regarding the health of the population based on blubber thickness—even if sampling occurs following standardized age categories and sampling seasons, variations related to unknown factors can bias the interpretation of the blubber thickness data. Studies in several seal species demonstrate that a qualitative and quantitative analysis of various parameters in and of the blubber have the potential to reveal further information about health and physiology like fatty acid composition and stratification (Walton and Pomeroy 2003; Guerrero et al., 2017), hormone levels and profiles and pollutants (Kershaw and Hall 2016; Troisi et al., 2020; Ogloff et al., 2022). Thus, further investigations including various analyses of some additional blubber parameters are strongly desired for a better evaluation of the overall health condition of individuals. Furthermore, we recommend that a subset of randomly selected individuals with detailed health information available is used to calibrate and further develop the current thresholds, or that different threshold are set for the Southern Baltic.

Among the three investigated species, the relationship of blubber thickness with time of the year was most pronounced in harbor porpoises, especially juveniles. Strong seasonal variations of blubber thickness have also been described for harbor porpoises living in a semi-open facility (Lockyer et al., 2003). Although topographical differences are described in the body distribution of blubber in harbor porpoises (Koopman, 1998; Lockyer et al., 2003; Rojano-Doñate et al., 2018), our results suggest that seasonal variations are similar for all body measurement sites, and hence that all the measurement points are useful for a relative indication of energy reserves. Although two distinct populations of harbor porpoises are recognized in the Baltic Sea, and their differing conservation status (i.e., the Baltic Proper population is listed as Critically Endangered by the IUCN and the Baltic Marine Environment Protection Commission [Hammond et al., 2008; HELCOM, 2013; ASCOBANS, 2016]), no separation between individuals from the two populations was performed in this study. This decision was based on providing a stronger basis for the analyses, with the final aim of providing an approach to the evaluation of blubber thickness in this species for future inclusion in the HELCOM core indicator system. This is by far the largest data set ever to be collected for blubber thickness in this area for these species. The data comprise hunted, bycaught and stranded animals. Among grey seals from Finland and Sweden, bycaught individuals displayed thinner blubber than hunted seals (Bäcklin et al., 2011; Kauhala et al., 2015) for unknown reasons as systematic health investigations are lacking on those animals. Future research should combine blubber thickness data and genetic data to infer differences among the two populations. The results from this study show that blubber thickness is an interesting metric in a wider system of indicators, but both intra- and inter-population variability need to be considered in the implementation of this metric to avoid bias in interpretations of population condition and trends.

In a large region like the Baltic Sea, inter-population baseline data need to be collated and verified for the entire geographical area prior to the development of region-wide thresholds. While the evaluation of the blubber thickness of individuals can provide information for an entire population, there needs to be adequate amounts of representative data across the region to ensure accurate assessments. Additionally, standardization of blubber thickness measurements throughout the Baltic Sea is mandatory for proper comparisons. Despite the enticing simplicity in terms of methodology, blubber thickness is not an adequate indicator of marine mammal health when used alone (Derous et al., 2020). Several drivers, both anthropogenic and natural environmental, can have an impact on individual health and jeopardize survival without causing immediate changes in the blubber thickness of the animal (e.g., acute vs chronic conditions). Deviations occurring between distinct years or more prolonged temporal trends, reflecting environmental changes (e.g., shifting foraging habitats, changing prey availability or shifts in prey distribution, regional climate changes resulting in different rates of blubber tissue production and storage) or biotic factors affecting blubber thickness dynamics in individuals (e.g., modified reproductive activity, disease prevalence and impact, stress induced by anthropogenic activities, different regional concentrations of pollutants, individual variability) are not solely be depicted by the evaluation of just one parameter.

Several previous studies have indicated that the health status of marine mammals is not related to blubber thickness. Infectious diseases can develop quickly, not being reflected in the blubber thickness or nutritional status of an individual (Siebert et al., 2001; Siebert et al., 2007; Bodewes et al., 2015; Siebert et al., 2020). In addition, the origin of an animal (e.g., by-caught, euthanized or stranded) does not necessarily reflect the health status of the individual, as by-caught animals display large variety of infectious diseases and stranded individuals can be traumatized by anthropogenic activities (Siebert et al., 2022). Therefore, standardized necropsies on the individuals from which blubber thickness values are derived are extremely important (IJsseldijk et al., 2019). Considerations regarding the health status of wild marine mammal populations need to incorporate indicators developed for each species where metrics are derived from complete individual inspections (i.e., full post-mortem examinations, clinical check-ups in live animals) originating from health monitoring programs that survey population health on a long-term basis. In this respect, a cooperative and standardized effort across borders is needed to enlarge data sets that could guarantee robust inferences regarding overall population status and important biological variations.

## Data Availability

The raw data supporting the conclusion of this article will be made available by the author, without undue reservation.
